# Transfer of Herb-Resistance Plasmid From *Escherichia coli* to *Staphylococcus aureus *Residing in the Human Urinary Tract

**DOI:** 10.5812/jjm.15056

**Published:** 2014-03-01

**Authors:** Yan Qing Tong, Bing Xin, Li Zhu

**Affiliations:** 1 Department of Nephrology, The First Affiliated Hospital to Changchun University of Chinese Medicine, Changchun, China; 2 Department of Microbiology, The First Affiliated Hospital to Changchun University of Chinese Medicine, Changchun, China

**Keywords:** *Escherichia coli*, Resistance, Plasmid

## Abstract

**Background:**

Plasmid transfer among bacteria provides a means for dissemination of resistance. Plasmid Analysis has made it possible to track plasmids that induce resistance in bacterial population.

**Objectives:**

To screen the presence of herb-resistance plasmid in *Escherichia coli* strains and determine the transferability of this resistance plasmid directly from *E. coli* to the Gram-positive, *Staphylococcus aureus*.

**Materials and Methods:**

The donor strain *E. coli* CP9 and recipient strain *S. aureus* RN450RF were isolated from UTI patients. *E. coli* CP9 was highly resistant to herbal concoction. Isolates of *S. aureus* RN450RF were fully susceptible. Total plasmid DNA was prepared and transferred into *E. coli* DH5α. Transconjugants were selected on agar plates containing serial dilutions of herbal concoction. Resistance plasmid was transferred to susceptible *S. aureus* RN450RFin triple replicas. The mating experiments were repeated twice.

**Results:**

The identified 45 kb herb-resistance plasmid could be transferred from *E. coli* CP9 isolates to *E. coli* DH5α. As a consequence *E. coli* DH5α transconjugant MIC increased from 0.0125 g/mL to 0.25 g/mL. The plasmid was easily transferred from *E. coli* CP9 strain to *S. aureus* RN450RF with a mean transfer rate of 1×10^-2 ^transconjugants/recipient. The *E. coli* donor and the *S. aureus* RN450RF transconjugant contained a plasmid of the same size, which was absent in the recipient before mating. Susceptibility testing showed that the *S. aureus* RN450RF transconjugant was resistant to herbal concoction.

**Conclusions:**

*E. coli* herb-resistance plasmid can replicate and be expressed in *S. aureus*.

## 1. Background

A large percentage of recurrent urinary tract infections (UTI) are caused by the same strain of bacteria as the initial infection (1). The high frequency of same-strain recurrences support the notion that a bacteria reservoir may exist in the affected individuals. Transfer of resistance plasmids among potentially pathogenic bacteria in the urinary tract results in multi-resistant bacteria production which might cause spreading infections to different organs and ultimately treatment failure.

Herbal medicine has been widely used for treatment of the UTIs. The herbal formulation used in this study was proved to have antimicrobial effects in our previous research (2, 3). The extensive use of herbal formulations has resulted in an increase in resistance of bacteria and is hypothesized to be the result of not only the selection and spread of resistant microorganisms, but also the intra- and inter-species transfer of resistance plasmids. Plasmid transfer among bacteria provides a means for dissemination of resistance. Analysis of plasmids has made it possible to track resistance plasmids in the bacterial population.

*Escherichia coli* remains an important cause of community-acquired UTIs in both developing and industrialized countries. *Staphylococcus aureus* is a relatively uncommon cause of UTI in the general population. Studies have shown that conjugation is a nonspecific process and accounts for most horizontal gene transfers between even remote bacterial species (4, 5).

## 2. Objectives

In this research the presence of plasmids in such strains was screened, and the transferability of plasmid resistance directly from *E. coli* to the Gram-positive, *S. aureus* was determined.

## 3. Materials and Methods

### 3.1. Bacterial Strains and Media

A donor strain *E. coli* CP9 was isolated from a patient with UTI. Its characteristics were as follows; β-hemolysis, O4/K54/H5 serotype, P pilus (class I Pap Gadhesin) and an aerobactin minus genotype. The recipient was a *S. aureus* strain RN450RF isolated from another patient with UTI. Its characteristics were as follows; strain derivation RN450; phenotypeα Phage-free, Rif^r^Fus^r^. Strains isolation was performed according to the standard laboratory protocols and strains were isolated from >10^5^ cfu of auropathogen per millilitre of midstream urine culture. After isolation, the bacteria were kept frozen at -70^o^C after the addition of 20% (v/v) glycerol and they were not sub-cultured more than twice before the investigation. Both donor and recipient strains had different morphologies.

The selective plate used was Luria–Bertani (LB) agar (Hao Ran Bio-Technology Co. China) with serial dilutions of herbal concoction added. The plates were incubated at 35^o^C for 24 hours, and the colonies were counted. Selected transconjugants were frozen at -40^o^C in 10% glycerol for further investigations.

### 3.2. Chinese Herbs and Susceptibility Test

The Chinese herbs were prepared with the following ratio: Tong Cao (Medulla Tetrapanacis) 4 : Hua Shi (Talcum) 20: Chi Shao (Radix PaeoniaeRubrae) 20 : Xiao Hui Xiang (Fructus Foeniculi Vulgaris) 30: Rou Gui (Cortex Cinnamomi) 30 : Li Zhi He (Semen Litchi) 30: TianKuiZi (Radix Semiaquilegiae) 30 : ZiHua Di Ding (Herba cum Rd ViolaeYedoensitis) 30: Qu Mai (HerbaDianthi) 40 : Ma Chi Xian (HerbaPortulacae) 60: Pu Gong Ying (Herbataraxaci) 60. The eleven crude drugs were mixed in 800 mL water (100^o^C for 30 minutes twice), leaving 100 mL of the liquor after decanting the mixture. It was then was centrifuged, filtered and sterilized with a solution of 0.5 g/mL as the drug concentration. Standard disc diffusion methodology (6) was used to test the concoction against strains.

The bacterial suspension was adjusted until the OD_620_ reached 0.4. The minimal inhibitory concentration (MIC) of herbal solution for strains was determined as follows: starting with the 0.5 g/mL concentration, eight serial dilutions were prepared in LB agar (1.0 g/mL, 0.5 g/mL, 0.2 g/mL, 0.1 g/mL, 0.05 g/mL, 0.025 g/mL, 0.0125 g/mL and 0.0 g/mL) and these concentrations were inoculated with the bacterial strain. The MIC was described as the lowest concentration of herbal solution that prevented obvious turbidity after incubation for 18 hours at 37^o^ C.

The *E. coli* strain ATCC 25922 was used as a quality control for validation of the MIC.

### 3.3. Plasmid Characterization

Total plasmid DNA was prepared (Plasmid Extraction Kit, Beijing TIANGEN) and transferred into *E. coli* DH5α (Invitrogen, Carlsbad, CA, USA). Transconjugants were selected on agar plates containing serial dilutions of herbal concoction after broth mating. Plasmid DNA was separated on a 0.7% agarose gel, stained with ethidium bromide, and visualized under UV-light.

### 3.4. Resistance Transfer

Experiments were performed using herb-resistant *E. coli* CP9 isolates as donors and susceptible *S. aureus* RN450RF as the recipient. *In vitro* mating was performed to investigate transferability of the resistance plasmid between the donor and recipient strains by Schjorring et al.’s method (7). Broth mating experiments were performed as follows; overnight cultures of donor and recipient strains were mixed with a ratio of 1:1, and the mixture was washed in saline (0.9% NaCl); an aliquot of 20 mL of the mixture was spotted on an LB plate incubated at 37^o^C for 24 hours and plated on selective plates. Selective plates contained serial dilutions of herbal concoction. All experiments were done in triplicates and the mating experiments were repeated twice.

## 4. Results

### 4.1. Susceptibility Testing of Strains

All isolates of strain *E. coli* CP9 were highly resistant to herbal concoction with MIC of 1.0 g/mL and zone diameter of 2 mm. All isolates of strain *S. aureus* RN450RF were fully susceptible to the herbal concoction tested (MIC of 0.025 g/mL and zone diameter of 11 mm). The strain *E. coli* DH5αMIC was 0.0125 g/mL with zone diameter of 12 mm.

### 4.2. Resistance Plasmid Patterns

The herbal resistance plasmid of the clinical strain *E. coli* CP9 was studied *in vitro*. *E. coli* CP9 was used as a donor and *E. coli* DH5α was used as a recipient. Transconjugants were detected on plates containing herbal concoction after broth mating. All transconjugants were tested against serial dilutions of herbal concoction and were found to be resistant to herbal concoction (MIC of 0.25 g/mL and zone diameter of 4 mm).

**Figure 1.**
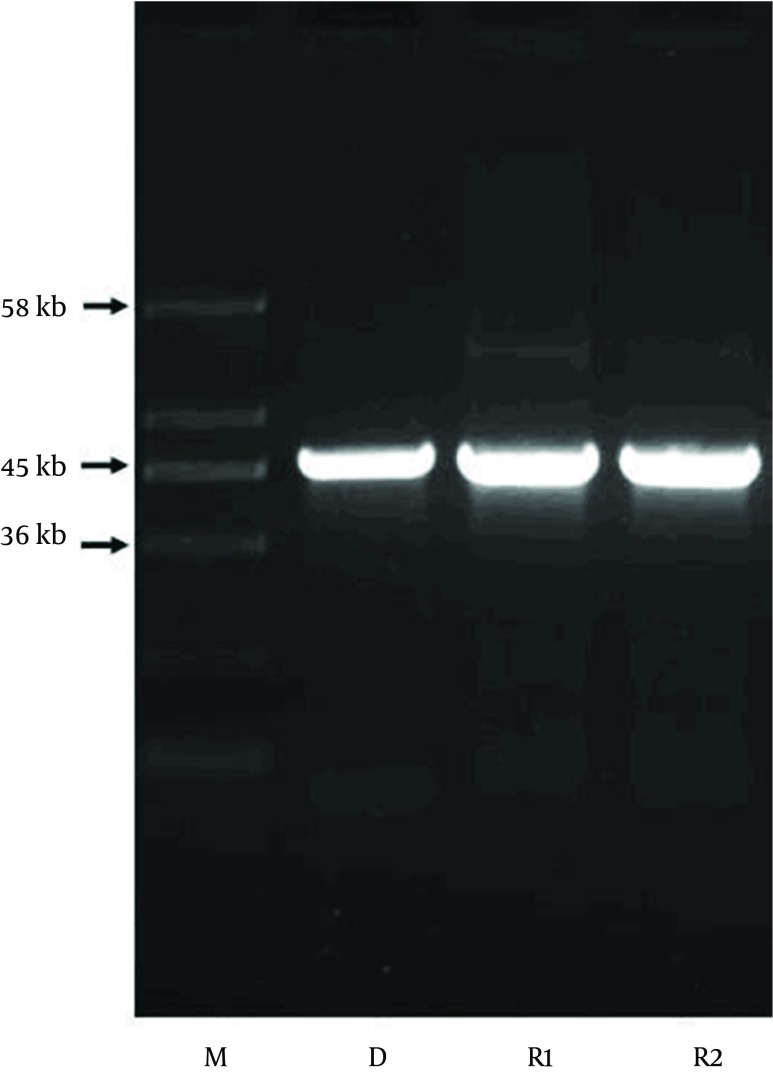
Resistance Plasmid Pattern Lane M, molecular size marker; lane D, plasmid fragment of *E. coli* CP9 as a donor; lane R1 and R2, trans conjugants of *E. coli* DH5α obtained using *E. coli* CP9 as a donor.

We examined plasmid similarity between strain *E. coli* CP9 and strain *E. coli* DH5α by restriction fragment length polymorphism analysis. Plasmid DNA fragments, 45 kb in size, were detected in the *E. coli* CP9, but were absent in *E. coli* DH5α before mating. After mating, the transconjugants of *E. coli* DH5α were seen to harbor this fragment (Figure 1) and exhibited resistance towards herbal concoction. Transconjugation experiments demonstrated that this plasmid fragment could be transferred from *E. coli* CP9 to *E. coli* DH5α isolates. The presence of this plasmid increased the MIC of herbal concoction from 0.0125 g/mL to 0.25 g/mL.

### 4.3. Transfer of Resistance

**Figure 2.**
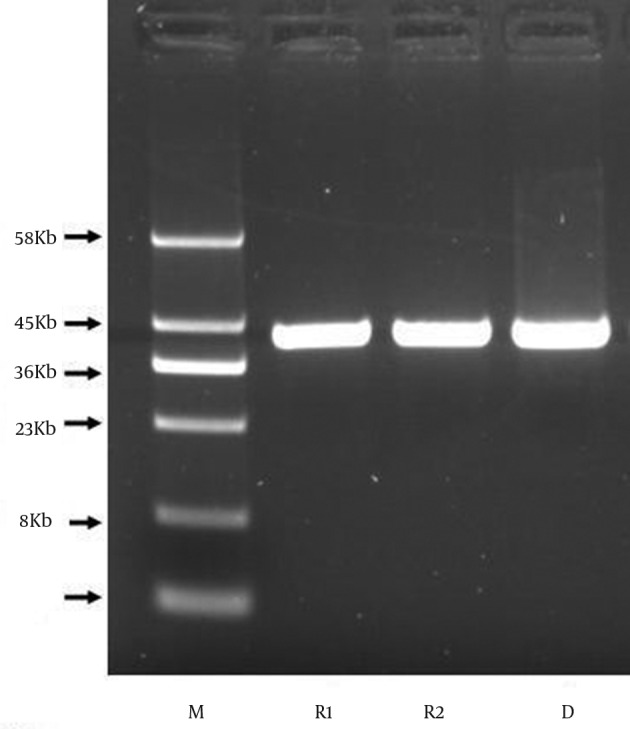
Transfer of Plasmid From *E. coli* CP9 to *S. aureus* Lane M, molecular size marker; lane D, plasmid from *E. coli* CP9 strain; lane R1 and R2, trans conjugants of *S*. *aureus* RN1801 obtained using *E. coli* CP9 strain as a donor.

*In vitro* broth mating showed that the identified plasmid was easily transferred from *E. coli* CP9 strain to *S. aureus* RN450RF with a mean transfer rate of 1×10^-2 ^transconjugants/recipient. Furthermore, after comparing the plasmid profiles, the *E. coli* donor and the transconjugant contained a plasmid of the same size (45 kb), which was absent in the recipient before mating (Figure 2). Susceptibility testing showed that the transconjugant was resistant to herbal concoction (MIC 0.125 g/mL, zone diameter 3 mm). This demonstrates that a 45 kb plasmid was transferred from the *E. coli* to the Gram-positive recipient, *S. aureus*. The plasmid persisted to the next generation of *S. aureus* which still showed resistance to herbal concoction.

## 5. Discussion

The convenience of the Chinese herbs use is well recognized. However, increased prescription of Chinese herbs for common infections such as UTIs will facilitate the emergence of Chinese herb-resistant strains. Although clinical strains resistant to Chinese herbs were isolated in our previous study, the resistance plasmid was not identified. Since Chinese herbs are currently used for UTIs, we felt it is appropriate to develop a stronger understanding of the existence of plasmid-mediated resistance and its transferability among heterologous bacteria.

In the present research, a herbal resistance plasmid was identified. The plasmid could replicate and express its genetic information (herbal resistance) in a remote new host. However, despite the herbal protective properties attributed to the plasmid, the underlying molecular mechanisms of these traits and the overall molecular biology of the plasmid are not understood.

As a large plasmid, multiple plasmids and genes for resistance varying in size may exist on the plasmid we identified. The questions about where these plasmids and genes originated and what purpose they served before being recruited to protect bacteria from Chinese herbs are to be answered. Postulating that genes originated on the chromosome of an organism occupying a human, veterinary or environmental reservoir genome sequences of bacteria species from a wide range of genera are to be screened by further research.

Most of the plasmids studied so far have a narrow host range. This study shows that the identified 45 kb plasmid can be transferred among heterologous bacteria residing in the human urinary tract, which indicates that the plasmid can replicate in a wider host range. Hence, we argue that the horizontal transfer of this resistance plasmid between *S. aureus* and *E. coli *occurred, while the two strains coexisted in the human urinary tract. *In vitro* transfer results cannot be directly extrapolated to the *in vivo* situation. Netherwood et al. (8) showed that *in vitro* methods, such as forced filter and liquid mating, underestimate the *in vivo* rates of gene transfer. This suggests that in order for the transfer to occur, high numbers of both the donor and the recipient should coincide in the urinary tract at the same time, as suggested in previous observations (9).

The extent to which a plasmid protects isolates against antibiotics has usually been examined by measuring the difference in antibiotic MICs for a strain with and without a plasmid. Unexpectedly, an increase in the herbal MIC was found. This plasmid increased herbal MIC 5-fold in a *S. aureus* transconjugant. Although this increase from baseline was not at the level designated to represent clinical resistance, the plasmid also facilitated the selection of higher-level resistance. In our study, donor bacteria originally harboring the plasmid, all exhibited higher levels of resistance to herbal concoction than the transconjugants, suggesting that additional mechanisms of herbal resistance frequently coexist with the plasmid identified.

MIC studies assess the effect of a resistance inducing gene on growth inhibition by an antimicrobial agent. There are other indices by which the effect of a resistance gene can be assessed. Bactericidal activities of antibiotics, which were evaluated by other time-kill studies (10, 11), will be examined by our future research.

Bacteria can acquire antimicrobial resistance when they are confronted with antibiotic selective pressures. The present study was performed without any selective pressure. The transfer frequency would probably have increased with the administration of antimicrobial agents. Of particular interest about this transmissible plasmid is that, in a single transfer event, may cause a major shift in the bacterial population dynamics by conferring resistance to Chinese herbs.

Transfer of any antimicrobial resistance genes is a threat which might result in limitation of treatment success and even treatment failures (12, 13). There is evidence that *S. aureus* is a primary urinary tract pathogen in long-term care patients. Using the CDC criteria for nosocomial infection to define UTIs, Muder et al. found that 4% of urinary tract originating bacteremia cases in this population were due to *S. aureus *(14). As a recipient of resistance plasmid, *S. aureus* transconjugant can induce bacteriuria which can lead to a subsequent invasive infection (5).

It is now understood that, concomitant with the expansion of the herbal medicine use, Gram-negative bacteria have assembled an arsenal of horizontally transmissible genetic elements that has facilitated the emergence of herbal resistance. However, clinical breakpoints have not yet been assessed in the context of the identified plasmid. With the discovery of the plasmid, we took an important step in the battle against resistance, but it is clear that the bacteria have had a head start.

The experiments reported here indicate that

(i) *S. aureus* strains can be transformed to herbal resistance with *E. coli* plasmids;
(ii) the transconjugants acquire plasmid;
(iii) this plasmid is indistinguishable from the *E. coli* plasmid by criteria of size. This evidence shows that *E. coli* plasmid can replicate and be expressed in *S. aureus*.
